# A second polymorph of 2,4,6-tris­(3,5-dimethyl-1*H*-pyrazol-1-yl)-1,3,5-triazine

**DOI:** 10.1107/S1600536812043255

**Published:** 2012-10-24

**Authors:** Seik Weng Ng

**Affiliations:** a Department of Chemistry, University of Malaya, 50603 Kuala Lumpur, Malaysia; b Chemistry Department, Faculty of Science, King Abdulaziz University, PO Box 80203 Jeddah, Saudi Arabia

## Abstract

The mol­ecule of the title compound, C_18_H_21_N_9_, is nearly planar, with the three pyrazole rings aligned at 2.40 (5), 9.27 (5) and 9.71 (5)° with respect to the triazine ring. The triazine ring is planar (r.m.s. deviation = 0.005 Å), the distortion from a hexa­gonal arrangement arising from the angles at the N [112.4 (1)–113.1 (1)°] and C [127.1 (1)–127.6 (1)°] atoms deviating from 120°. The crystal studied was an inversion twin.

## Related literature

For another *Pna*2_1_ polymorph, see: Guerrero *et al.* (2003 ▶). For a discussion of the determination of the absolute parameter, see: Flack & Bernardinelli (2000 ▶); Hooft *et al.* (2008 ▶); Spek (2009 ▶).
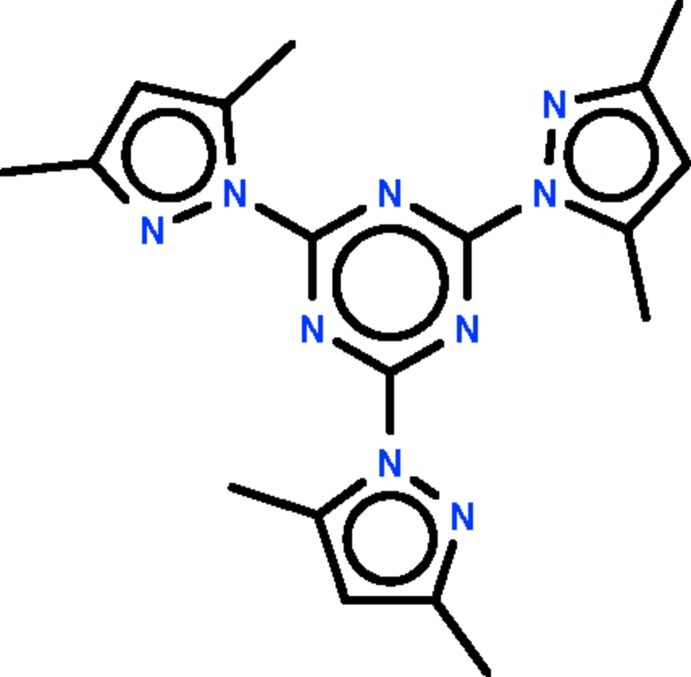


## Experimental

### 

#### Crystal data

C_18_H_21_N_9_
*M* *_r_* = 363.44
Orthorhombic, 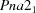

*a* = 7.1840 (1) Å
*b* = 12.5079 (1) Å
*c* = 19.9527 (1) Å
*V* = 1792.89 (3) Å^3^
*Z* = 4
Cu *K*α radiation
μ = 0.71 mm^−1^
*T* = 100 K
0.30 × 0.25 × 0.20 mm

#### Data collection

Agilent SuperNova Dual diffractometer with an Atlas CCD detector
Absorption correction: multi-scan (*CrysAlis PRO*; Agilent, 2012 ▶) *T*_min_ = 0.815, *T*_max_ = 0.871
33189 measured reflections
3744 independent reflections
3740 reflections with *I* > 2σ(*I*)
*R*_int_ = 0.016

#### Refinement

*R*[*F* ^2^ > 2σ(*F* ^2^)] = 0.028
*wR*(*F* ^2^) = 0.079
*S* = 1.06
3744 reflections
250 parameters
1 restraint
H-atom parameters constrained
Δρ_max_ = 0.17 e Å^−3^
Δρ_min_ = −0.23 e Å^−3^
Absolute structure: Flack (1983 ▶), 1813 Friedel pairs
Flack parameter: 0.51 (19)

### 

Data collection: *CrysAlis PRO* (Agilent, 2012 ▶); cell refinement: *CrysAlis PRO*; data reduction: *CrysAlis PRO*; program(s) used to solve structure: *SHELXS97* (Sheldrick, 2008 ▶); program(s) used to refine structure: *SHELXL97* (Sheldrick, 2008 ▶); molecular graphics: *X-SEED* (Barbour, 2001 ▶); software used to prepare material for publication: *publCIF* (Westrip, 2010 ▶).

## Supplementary Material

Crystal structure: contains datablock(s) global, I. DOI: 10.1107/S1600536812043255/zs2237sup1.cif

Structure factors: contains datablock(s) I. DOI: 10.1107/S1600536812043255/zs2237Isup2.hkl

Supplementary material file. DOI: 10.1107/S1600536812043255/zs2237Isup3.cml

Additional supplementary materials:  crystallographic information; 3D view; checkCIF report

## Figures and Tables

**Fig. 1.**
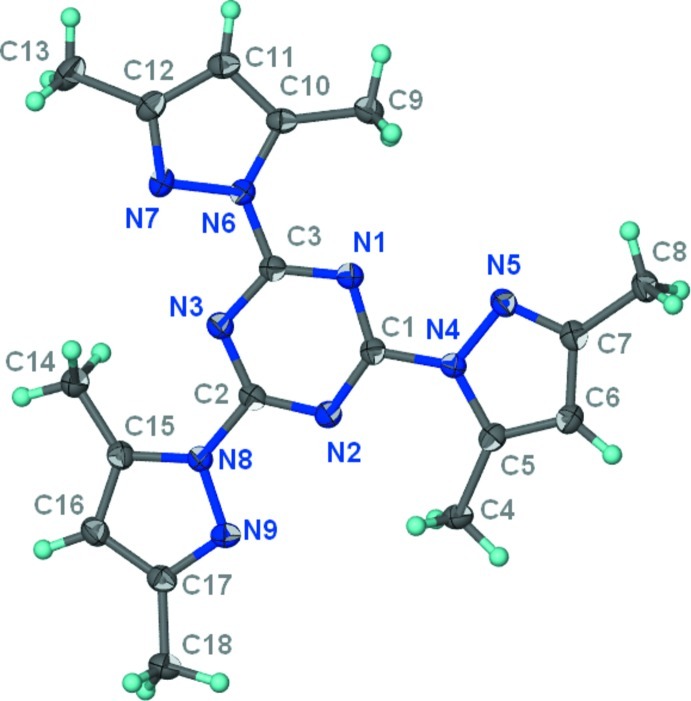
Thermal ellipsoid plot (Barbour, 2001) of C18H21N9 at the 70% probability level. Hydrogen atoms are drawn as spheres of arbitrary radius.

## supplementary crystallographic information

### Comment

Tris(3,5-dimethylpyrazol-1-yl)-1,3,5-triazine (Scheme I) was reported as being orthorhombic in the *Pna*2_1_ space group [7.941 (1), 12.555 (1), 18.901 (1) Å] (Guerrero *et al.*, 2003). For organic compounds belonging to noncentrosymmetric space groups, Friedel pairs are generally merged so that the absolute structure is not determined, particularly if the diffractometer measurements are of average quality.

A different polymorph that belongs to the same space group has been found in the present study. Because the diffraction measurements are of exceptionally high quality, the Flack parameter (Flack, 1983) could be refined. As this parameter refined to 0.5 (2), the structure (Fig. 1) is then interpreted as being a racemic twin. The somewhat more reliable Hooft value of 0.48 (3), with the smaller standard uncertainty, indicates that the absolute structure had probably been determined correctly.

The molecule of the title compound is nearly planar with the three pyrazole rings aligned at 2.40 (5), 9.27 (5) and 9.71 (5)° with respect to the triazine ring. The triazine ring is planar (r.m.s. deviation 0.005Å), the distortion from a hexagonal arrangement arising from the angles at the nitrogen [112.4 (1) to 113.1 (1) °] and carbon [127.1 (1) to 127.6 (1)°] atoms deviating from 120°.

### Experimental

3,5-Dimethylpyrazole (0.77 g, 8 mmol) and sodium hydride (0.38 g, 16 mmol) were added to a solution of cyanuric chloride (0.74 g, 4 mmol) dissolved in dry THF (40 ml). The mixture was stirred for 20 h. The THF was removed under reduced pressure and water was added. The organic compound was extracted with dichloromethane (3 × 15 ml). The organic layer was dried over magnesium sulfate. The solvent was evaporated and the product purified on silica gel. Colorless crystals were obtained upon recrystalization from a hexane-dichloromethane (1:1) mixture in 60% yield.

### Refinement

Carbon-bound H-atoms were placed in calculated positions [C–H 0.95 to 0.98 Å, *U*_iso_(H) 1.2 to 1.5 *U*_eq_(C)] and were included in the refinement in the riding model approximation. The absolute structure parameter *y* was calculated from Bayesian statistics (Hooft *et al.*, 2008) by using *PLATON* (Spek, 2009). The Friedel coverage was 99%; the Hooft value of 0.48 (3) indicates that the absolute structure had probably been determined correctly. The uncertainty in the absolute structure parameter was based on set criteria (Flack & Bernardinelli, 2000).

### Crystal data

**Table:**

C_18_H_21_N_9_	*F*(000) = 768
*M**_r_* = 363.44	*D*_x_ = 1.347 Mg m^−^^3^
Orthorhombic, *P**n**a*2_1_	Cu *K*α radiation, λ = 1.54184 Å
Hall symbol: P 2c -2n	Cell parameters from 23687 reflections
*a* = 7.1840 (1) Å	θ = 3.5–76.5°
*b* = 12.5079 (1) Å	µ = 0.71 mm^−^^1^
*c* = 19.9527 (1) Å	*T* = 100 K
*V* = 1792.89 (3) Å^3^	Prism, colorless
*Z* = 4	0.30 × 0.25 × 0.20 mm

### Data collection

**Table:**

Agilent SuperNova Dual diffractometer with an Atlas CCD detector	3744 independent reflections
Radiation source: SuperNova (Cu) X-ray Source	3740 reflections with *I* > 2σ(*I*)
Mirror monochromator	*R*_int_ = 0.016
Detector resolution: 10.4041 pixels mm^-1^	θ_max_ = 76.7°, θ_min_ = 4.2°
ω scans	*h* = −9→7
Absorption correction: multi-scan (*CrysAlis PRO*; Agilent, 2012)	*k* = −15→15
*T*_min_ = 0.815, *T*_max_ = 0.871	*l* = −25→25
33189 measured reflections	

### Refinement

**Table:**

Refinement on *F*^2^	Secondary atom site location: difference Fourier map
Least-squares matrix: full	Hydrogen site location: inferred from neighbouring sites
*R*[*F*^2^ > 2σ(*F*^2^)] = 0.028	H-atom parameters constrained
*wR*(*F*^2^) = 0.079	*w* = 1/[σ^2^(*F*_o_^2^) + (0.0643*P*)^2^ + 0.1472*P*] where *P* = (*F*_o_^2^ + 2*F*_c_^2^)/3
*S* = 1.06	(Δ/σ)_max_ = 0.001
3744 reflections	Δρ_max_ = 0.17 e Å^−^^3^
250 parameters	Δρ_min_ = −0.23 e Å^−^^3^
1 restraint	Absolute structure: Flack (1983), 1813 Friedel pairs
Primary atom site location: structure-invariant direct methods	Flack parameter: 0.51 (19)

### Fractional atomic coordinates and isotropic or equivalent isotropic displacement parameters (Å^2^)

**Table:**

	*x*	*y*	*z*	*U*_iso_*/*U*_eq_	
N1	0.64690 (14)	0.33516 (7)	0.49998 (4)	0.01670 (18)	
N2	0.76115 (13)	0.35089 (7)	0.61216 (5)	0.01684 (18)	
N3	0.70433 (13)	0.50706 (7)	0.54716 (5)	0.01692 (19)	
N4	0.70786 (13)	0.18485 (7)	0.56534 (4)	0.01575 (19)	
N5	0.65449 (13)	0.12198 (8)	0.51162 (5)	0.01787 (19)	
N6	0.58836 (13)	0.49074 (7)	0.43969 (4)	0.01651 (19)	
N7	0.60689 (13)	0.60069 (7)	0.43401 (5)	0.01858 (19)	
N8	0.81077 (13)	0.52146 (7)	0.65568 (4)	0.01588 (18)	
N9	0.89652 (12)	0.47659 (7)	0.71095 (5)	0.01760 (19)	
C1	0.70516 (15)	0.29617 (8)	0.55866 (6)	0.0150 (2)	
C2	0.75663 (14)	0.45598 (9)	0.60231 (5)	0.0154 (2)	
C3	0.64950 (15)	0.44148 (8)	0.49846 (5)	0.0153 (2)	
C4	0.82309 (17)	0.16382 (9)	0.68709 (5)	0.0204 (2)	
H4A	0.8586	0.1033	0.7155	0.031*	
H4B	0.7198	0.2026	0.7080	0.031*	
H4C	0.9297	0.2119	0.6818	0.031*	
C5	0.76404 (15)	0.12363 (9)	0.62003 (6)	0.0170 (2)	
C6	0.74711 (16)	0.01995 (9)	0.59952 (6)	0.0194 (2)	
H6	0.7756	−0.0421	0.6250	0.023*	
C7	0.67861 (14)	0.02291 (9)	0.53266 (6)	0.0176 (2)	
C8	0.63441 (17)	−0.07007 (9)	0.48831 (6)	0.0212 (2)	
H8A	0.6041	−0.0442	0.4432	0.032*	
H8B	0.5277	−0.1092	0.5066	0.032*	
H8C	0.7424	−0.1178	0.4860	0.032*	
C9	0.44991 (16)	0.33159 (9)	0.37583 (6)	0.0205 (2)	
H9A	0.3880	0.3215	0.3325	0.031*	
H9B	0.3669	0.3082	0.4119	0.031*	
H9C	0.5646	0.2892	0.3770	0.031*	
C10	0.49607 (15)	0.44699 (9)	0.38503 (5)	0.0170 (2)	
C11	0.45694 (16)	0.53202 (9)	0.34406 (6)	0.0194 (2)	
H11	0.3947	0.5295	0.3021	0.023*	
C12	0.52746 (15)	0.62463 (9)	0.37652 (6)	0.0191 (2)	
C13	0.51606 (18)	0.73881 (10)	0.35422 (7)	0.0257 (2)	
H13A	0.6340	0.7750	0.3640	0.039*	
H13B	0.4147	0.7748	0.3782	0.039*	
H13C	0.4920	0.7414	0.3059	0.039*	
C14	0.71358 (17)	0.70472 (9)	0.60992 (6)	0.0230 (2)	
H14A	0.7102	0.7776	0.6280	0.035*	
H14B	0.5864	0.6805	0.6007	0.035*	
H14C	0.7862	0.7039	0.5683	0.035*	
C15	0.80173 (15)	0.63183 (8)	0.65987 (6)	0.0179 (2)	
C16	0.88424 (16)	0.65728 (9)	0.71970 (6)	0.0204 (2)	
H16	0.9001	0.7269	0.7379	0.024*	
C17	0.94152 (15)	0.55897 (9)	0.74920 (5)	0.0180 (2)	
C18	1.03930 (18)	0.54346 (9)	0.81452 (6)	0.0236 (2)	
H18A	1.0544	0.4668	0.8232	0.035*	
H18B	0.9657	0.5758	0.8506	0.035*	
H18C	1.1620	0.5776	0.8126	0.035*	

### Atomic displacement parameters (Å^2^)

**Table:**

	*U*^11^	*U*^22^	*U*^33^	*U*^12^	*U*^13^	*U*^23^
N1	0.0178 (4)	0.0167 (4)	0.0155 (4)	−0.0006 (3)	−0.0008 (3)	0.0003 (3)
N2	0.0178 (4)	0.0169 (4)	0.0158 (4)	−0.0008 (3)	0.0006 (3)	0.0006 (3)
N3	0.0182 (4)	0.0165 (4)	0.0160 (5)	−0.0006 (3)	−0.0001 (3)	−0.0009 (3)
N4	0.0180 (4)	0.0157 (4)	0.0136 (4)	−0.0008 (3)	−0.0008 (3)	−0.0003 (3)
N5	0.0201 (4)	0.0171 (4)	0.0164 (4)	−0.0021 (3)	0.0009 (3)	−0.0022 (3)
N6	0.0182 (4)	0.0155 (4)	0.0158 (4)	−0.0013 (3)	−0.0010 (4)	0.0012 (4)
N7	0.0214 (4)	0.0147 (4)	0.0197 (4)	−0.0003 (3)	−0.0001 (4)	0.0027 (3)
N8	0.0181 (4)	0.0148 (4)	0.0147 (4)	−0.0004 (3)	−0.0004 (3)	−0.0007 (3)
N9	0.0193 (4)	0.0204 (4)	0.0131 (4)	0.0001 (3)	−0.0001 (3)	0.0004 (3)
C1	0.0140 (4)	0.0154 (5)	0.0156 (4)	−0.0014 (4)	0.0021 (3)	0.0006 (4)
C2	0.0135 (5)	0.0183 (5)	0.0144 (5)	−0.0017 (4)	0.0017 (4)	−0.0012 (4)
C3	0.0139 (4)	0.0173 (5)	0.0146 (5)	−0.0013 (4)	0.0006 (3)	0.0008 (4)
C4	0.0241 (5)	0.0214 (5)	0.0157 (5)	0.0016 (4)	−0.0032 (4)	0.0014 (4)
C5	0.0163 (5)	0.0176 (5)	0.0171 (5)	0.0007 (4)	0.0023 (4)	0.0022 (4)
C6	0.0216 (5)	0.0170 (5)	0.0195 (5)	0.0008 (4)	0.0015 (4)	0.0022 (4)
C7	0.0171 (4)	0.0181 (5)	0.0178 (5)	−0.0013 (4)	0.0037 (4)	−0.0009 (4)
C8	0.0273 (6)	0.0170 (5)	0.0193 (5)	−0.0016 (4)	0.0035 (4)	−0.0027 (4)
C9	0.0238 (5)	0.0212 (5)	0.0166 (5)	−0.0024 (4)	−0.0019 (4)	−0.0013 (4)
C10	0.0149 (5)	0.0222 (5)	0.0138 (5)	0.0001 (4)	0.0004 (4)	0.0003 (4)
C11	0.0168 (5)	0.0250 (5)	0.0164 (5)	−0.0004 (4)	0.0002 (4)	0.0020 (4)
C12	0.0161 (5)	0.0200 (5)	0.0212 (5)	0.0007 (4)	0.0019 (4)	0.0049 (4)
C13	0.0241 (6)	0.0219 (5)	0.0313 (6)	0.0020 (4)	−0.0015 (5)	0.0098 (4)
C14	0.0300 (6)	0.0173 (5)	0.0219 (5)	0.0023 (4)	−0.0041 (5)	−0.0005 (4)
C15	0.0196 (5)	0.0171 (5)	0.0168 (5)	−0.0014 (4)	0.0019 (4)	−0.0009 (4)
C16	0.0246 (5)	0.0191 (5)	0.0176 (5)	−0.0019 (4)	0.0004 (4)	−0.0031 (4)
C17	0.0176 (5)	0.0213 (5)	0.0151 (5)	−0.0020 (4)	0.0014 (4)	−0.0012 (4)
C18	0.0285 (6)	0.0250 (5)	0.0175 (5)	−0.0007 (5)	−0.0052 (5)	−0.0018 (4)

### Geometric parameters (Å, º)

**Table:**

N1—C3	1.3303 (13)	C8—H8A	0.9800
N1—C1	1.3356 (14)	C8—H8B	0.9800
N2—C2	1.3295 (14)	C8—H8C	0.9800
N2—C1	1.3302 (14)	C9—C10	1.4923 (15)
N3—C2	1.3268 (14)	C9—H9A	0.9800
N3—C3	1.3312 (14)	C9—H9B	0.9800
N4—N5	1.3836 (13)	C9—H9C	0.9800
N4—C5	1.3929 (14)	C10—C11	1.3705 (15)
N4—C1	1.3989 (13)	C11—C12	1.4206 (16)
N5—C7	1.3197 (14)	C11—H11	0.9500
N6—N7	1.3863 (11)	C12—C13	1.4980 (14)
N6—C10	1.3887 (14)	C13—H13A	0.9800
N6—C3	1.3957 (13)	C13—H13B	0.9800
N7—C12	1.3157 (15)	C13—H13C	0.9800
N8—N9	1.3822 (12)	C14—C15	1.4918 (15)
N8—C15	1.3846 (13)	C14—H14A	0.9800
N8—C2	1.3985 (13)	C14—H14B	0.9800
N9—C17	1.3225 (14)	C14—H14C	0.9800
C4—C5	1.4909 (15)	C15—C16	1.3703 (15)
C4—H4A	0.9800	C16—C17	1.4240 (15)
C4—H4B	0.9800	C16—H16	0.9500
C4—H4C	0.9800	C17—C18	1.4931 (15)
C5—C6	1.3653 (15)	C18—H18A	0.9800
C6—C7	1.4225 (16)	C18—H18B	0.9800
C6—H6	0.9500	C18—H18C	0.9800
C7—C8	1.4954 (15)		
			
C3—N1—C1	112.37 (9)	H8B—C8—H8C	109.5
C2—N2—C1	112.51 (9)	C10—C9—H9A	109.5
C2—N3—C3	113.11 (9)	C10—C9—H9B	109.5
N5—N4—C5	112.01 (9)	H9A—C9—H9B	109.5
N5—N4—C1	119.22 (9)	C10—C9—H9C	109.5
C5—N4—C1	128.74 (10)	H9A—C9—H9C	109.5
C7—N5—N4	104.53 (9)	H9B—C9—H9C	109.5
N7—N6—C10	111.88 (8)	C11—C10—N6	105.10 (9)
N7—N6—C3	118.44 (9)	C11—C10—C9	129.18 (11)
C10—N6—C3	129.51 (9)	N6—C10—C9	125.72 (9)
C12—N7—N6	104.79 (9)	C10—C11—C12	106.73 (10)
N9—N8—C15	112.18 (8)	C10—C11—H11	126.6
N9—N8—C2	119.58 (8)	C12—C11—H11	126.6
C15—N8—C2	128.08 (9)	N7—C12—C11	111.50 (10)
C17—N9—N8	104.65 (9)	N7—C12—C13	119.98 (10)
N2—C1—N1	127.61 (10)	C11—C12—C13	128.49 (11)
N2—C1—N4	115.56 (10)	C12—C13—H13A	109.5
N1—C1—N4	116.83 (9)	C12—C13—H13B	109.5
N3—C2—N2	127.27 (10)	H13A—C13—H13B	109.5
N3—C2—N8	115.36 (9)	C12—C13—H13C	109.5
N2—C2—N8	117.37 (9)	H13A—C13—H13C	109.5
N1—C3—N3	127.12 (10)	H13B—C13—H13C	109.5
N1—C3—N6	117.13 (9)	C15—C14—H14A	109.5
N3—C3—N6	115.75 (9)	C15—C14—H14B	109.5
C5—C4—H4A	109.5	H14A—C14—H14B	109.5
C5—C4—H4B	109.5	C15—C14—H14C	109.5
H4A—C4—H4B	109.5	H14A—C14—H14C	109.5
C5—C4—H4C	109.5	H14B—C14—H14C	109.5
H4A—C4—H4C	109.5	C16—C15—N8	105.30 (9)
H4B—C4—H4C	109.5	C16—C15—C14	128.59 (10)
C6—C5—N4	105.16 (10)	N8—C15—C14	126.08 (10)
C6—C5—C4	127.92 (11)	C15—C16—C17	106.53 (10)
N4—C5—C4	126.89 (10)	C15—C16—H16	126.7
C5—C6—C7	106.69 (10)	C17—C16—H16	126.7
C5—C6—H6	126.7	N9—C17—C16	111.33 (10)
C7—C6—H6	126.7	N9—C17—C18	121.16 (10)
N5—C7—C6	111.60 (10)	C16—C17—C18	127.51 (10)
N5—C7—C8	120.94 (10)	C17—C18—H18A	109.5
C6—C7—C8	127.46 (10)	C17—C18—H18B	109.5
C7—C8—H8A	109.5	H18A—C18—H18B	109.5
C7—C8—H8B	109.5	C17—C18—H18C	109.5
H8A—C8—H8B	109.5	H18A—C18—H18C	109.5
C7—C8—H8C	109.5	H18B—C18—H18C	109.5
H8A—C8—H8C	109.5		
			
C5—N4—N5—C7	−0.42 (12)	N5—N4—C5—C6	0.66 (12)
C1—N4—N5—C7	177.77 (9)	C1—N4—C5—C6	−177.31 (10)
C10—N6—N7—C12	−0.13 (11)	N5—N4—C5—C4	−177.36 (10)
C3—N6—N7—C12	−175.89 (9)	C1—N4—C5—C4	4.66 (18)
C15—N8—N9—C17	−0.33 (11)	N4—C5—C6—C7	−0.62 (11)
C2—N8—N9—C17	175.53 (9)	C4—C5—C6—C7	177.38 (11)
C2—N2—C1—N1	−0.66 (16)	N4—N5—C7—C6	0.01 (11)
C2—N2—C1—N4	179.50 (9)	N4—N5—C7—C8	179.63 (9)
C3—N1—C1—N2	0.23 (16)	C5—C6—C7—N5	0.40 (12)
C3—N1—C1—N4	−179.92 (9)	C5—C6—C7—C8	−179.20 (11)
N5—N4—C1—N2	−178.99 (9)	N7—N6—C10—C11	0.30 (12)
C5—N4—C1—N2	−1.14 (16)	C3—N6—C10—C11	175.47 (10)
N5—N4—C1—N1	1.15 (14)	N7—N6—C10—C9	−178.78 (10)
C5—N4—C1—N1	179.00 (10)	C3—N6—C10—C9	−3.62 (18)
C3—N3—C2—N2	0.98 (15)	N6—C10—C11—C12	−0.34 (12)
C3—N3—C2—N8	−178.34 (9)	C9—C10—C11—C12	178.70 (11)
C1—N2—C2—N3	−0.01 (15)	N6—N7—C12—C11	−0.09 (12)
C1—N2—C2—N8	179.29 (9)	N6—N7—C12—C13	178.25 (10)
N9—N8—C2—N3	−169.23 (9)	C10—C11—C12—N7	0.28 (13)
C15—N8—C2—N3	5.90 (15)	C10—C11—C12—C13	−177.89 (11)
N9—N8—C2—N2	11.38 (14)	N9—N8—C15—C16	−0.05 (11)
C15—N8—C2—N2	−173.49 (10)	C2—N8—C15—C16	−175.48 (10)
C1—N1—C3—N3	0.97 (15)	N9—N8—C15—C14	−178.12 (10)
C1—N1—C3—N6	−179.01 (9)	C2—N8—C15—C14	6.46 (17)
C2—N3—C3—N1	−1.51 (15)	N8—C15—C16—C17	0.39 (12)
C2—N3—C3—N6	178.46 (9)	C14—C15—C16—C17	178.39 (11)
N7—N6—C3—N1	−173.61 (9)	N8—N9—C17—C16	0.58 (11)
C10—N6—C3—N1	11.49 (16)	N8—N9—C17—C18	−179.66 (10)
N7—N6—C3—N3	6.41 (14)	C15—C16—C17—N9	−0.64 (13)
C10—N6—C3—N3	−168.49 (10)	C15—C16—C17—C18	179.63 (11)
